# Reversible and Irreversible Effects of Electroporation on Contractility and Calcium Homeostasis in Isolated Cardiac Ventricular Myocytes

**DOI:** 10.1161/CIRCEP.122.011131

**Published:** 2022-10-28

**Authors:** Sébastien Chaigne, Daniel C. Sigg, Mark T. Stewart, Mélèze Hocini, Tina Batista Napotnik, Damijan Miklavčič, Olivier Bernus, David Benoist

**Affiliations:** IHU LIRYC Electrophysiology and Heart Modeling Institute, Fondation Bordeaux Université, Bordeaux, France (S.C., M.H., O.B., D.B.).; University of Bordeaux, Inserm, CRCTB U1045, Bordeaux, France (S.C., M.H., O.B., D.B.).; Medtronic Cardiac Ablation Solutions, Minneapolis, MN (D.C.S., M.T.S.).; CHU de Bordeaux, Hôpital Cardiologique Haut-Lévêque, Pessac, France (S.C., M.H.).; Faculty of Electrical Engineering, University of Ljubljana, Ljubljana, Slovenia (T.B.N., D.M.).

**Keywords:** calcium, electroporation, homeostasis, myocytes, cardiac, sacromere

## Abstract

Irreversible electroporation is an energy form utilizing high-voltage pulsed electric field, leading to cellular homeostasis disruption and cell death. Recently, irreversible electroporation has shown promising results for the treatment of cardiac arrhythmias. However, reversible and irreversible effects of pulsed electric field on cardiac myocytes remain poorly understood. Here, we evaluated the influence of a monophasic single electric pulse (EP) on the contractility, Ca^2+^ homeostasis and recovery of cardiac myocytes.

**Methods:**

Isolated rat left ventricular myocytes were electroporated using single monophasic EP of different durations and voltages. Sarcomere length and intracellular Ca^2+^ were simultaneously monitored for up to 20 minutes after EP application in Fura-2 loaded left ventricular myocytes. Lethal voltage thresholds were determined using 100 µs and 10 ms pulses and by discriminating cell orientation with respect to the electric field.

**Results:**

Electroporation led to an immediate increase in intracellular Ca^2+^ which was dependent upon the voltage delivered to the cell. Intermediate-voltage EP (140 V, 100 µs) increased sarcomere shortening, Ca^2+^ transient amplitude, and diastolic Ca^2+^ level measured 1 minute post-EP. Although sarcomere shortening returned to pre-EP level within 5 minutes, Ca^2+^ transient amplitude decreased further below pre-EP level and diastolic Ca^2+^ level remained elevated within 20 minutes post-EP. Spontaneous contractions were observed after sublethal EP application but their frequency decreased progressively within 20 minutes. Lethal EP voltage threshold was lower in myocytes oriented perpendicular than parallel to the electric field using 100 µs pulses while an opposite effect was found using 10 ms pulses.

**Conclusions:**

Sublethal EP affected rat left ventricular myocytes contractility and disrupted Ca^2+^ homeostasis as a function of the EP voltage. Moreover, EP-induced lethality was preceded by a large increase in intracellular Ca^2+^ and was dependent upon the EP duration, amplitude and left ventricular myocytes orientation with respect to the electric field. These findings provide new insights into the effect of pulsed electric field on cardiac myocytes.

**What Is Known?**

- Electroporation is a promising novel energy modality for safe, efficient cardiac arrhythmia ablation.
- Pulsed-electrical fields may induce both reversible and irreversible electroporation when applied to the myocardium but these effects remain poorly characterized.

**What the Study Adds**

- Irreversible electroporation is preceded by a large increase in intracellular Ca^2+^ and depends on the pulse amplitude and duration.
- Cell orientation with respect to the electrical field is a determinant of myocyte’s lethality induced by electroporation.
- Sublethal electroporation is associated with perturbations in intracellular Ca^2+^ and contractility in isolated myocytes.

Electroporation is a phenomenon which is associated with transient increase in membrane permeability and is achieved by cell exposure to high-voltage electric pulses (EPs). When cells are exposed to electric field strengths and duration that exceed a threshold, the increase in membrane permeability and disruption of cell homeostasis will lead to cell death. This is described as irreversible electroporation (IRE). Cells following exposure to electric fields below the IRE threshold may however regain homeostasis and survive.^1^ Technologies leveraging pulsed electric field (PEF) to achieve reversible and IRE have been developed in a broad range of medical specialties including oncology,^2–6^ neurology,^7,8,9,10^ dermatology^11,12^ and most recently, cardiology.^13,14,15,16^ Specifically, the use of PEF to ablate aberrant cardiac tissues via IRE is rapidly emerging as a promising energy modality in the treatment of cardiac arrhythmias. Traditional cardiac ablation techniques involve the use of thermal energy modalities such as radiofrequency ablation and cryoablation.^17,18^ One of the most common procedures within cardiac catheter ablation is pulmonary vein isolation. Pulmonary vein isolation aims to isolate atrial fibrillation triggers located in the pulmonary veins via selective ablation of this area. Although pulmonary vein isolation using thermal ablation technologies are approved and generally effective and safe clinical procedures for the treatment of atrial fibrillation, they are still plagued by both safety and efficacy issues. For example, thermal-based energy modalities used for pulmonary vein isolation procedures may cause collateral and detrimental tissue damage leading to pulmonary vein stenosis, phrenic nerve paralysis, esophageal fistulas, or bronchial damage. Incomplete lesion formation can result in pulmonary vein reconnection and arrhythmia recurrence.^19^

Pulsed field ablation (PFA) is a term used to describe the use of IRE to ablate cardiac tissues for the treatment of cardiac arrhythmias. PFA utilizes hundreds to thousands of volts applied to electrodes on a catheter either in a monopolar or bipolar fashion/mode to induce cell death via membrane hyperpermeabilization mechanisms. PFA holds great promise and potential advantages over traditional energy modalities and may be able to alleviate/mitigate limitations of the thermal-based ablation modalities including collateral damage. It has been hypothesized that cardiac cells may be more sensitive to IRE than other cell types, such as nerve cells or smooth muscle cells, and thus less collateral injury is expected with PFA. This hypothesis is also supported by absence of side effects in preclinical as well as clinical studies.^14,20–23^ For example, in a recent preclinical study on PFA, a significantly reduced risk of pulmonary vein stenosis was reported in comparison to radiofrequency in a canine model.^24^ Radiofrequency ablations resulted in vagus nerve, esophageal, and lung injury, whereas this was not observed with PFA.^,14,24^ Although mechanisms of cell death after EP are complex,^25^ cell death mechanisms (and dynamics) of cardiac cells in response to PEF are not well understood. That said, it is generally known in the field of IRE that cell death is a function of the electric field.^26^ Because the ultimate goal of PFA is IRE, the application of high voltages to membranes may also induce reversible effects.^1^ Reversible electroporation is inevitable in vivo given the electric field distribution around electrodes.^,27,28^ Considering that electric field strength drops as the distance from the electrodes increases, it can be expected that a volume exists adjacent to the electrodes above the IRE threshold where all cells will die. Surrounding that ablated volume, there may be a region of tissue where transient or reversible effects will be induced. In this context, our understanding of the reversible and IRE effects on cardiac cellular function related to PEF application is desperately needed. In nerves, electrode proximity and voltage (field strength) can dose-dependently increase neural stunning times of paced phrenic nerves after PFA application.^29^ Previous defibrillation research has demonstrated transient effects on cardiac cell permeability in the early 2000s, such (electrical) stunning phenomenon has not been described in the context of PEF aimed to ablate cardiac tissue.^30,31^ It is possible that reversibly effected, that is “(electrically) stunned” zones could be observed clinically and would be interpreted as acute procedural success. But if such stunned zones recover, the ablation therapy could fail due to incomplete lesion formation/electrical isolation with subsequent pulmonary vein reconnection. Moreover, the orientation of elongated cells in electric field affects EP,^32,33,34,35,36,37,38,39^ which can play a significant role in ablating heart tissue where structure (position of cells) and function are strongly connected.

Due to the central role of Ca^2+^ in excitation-contraction coupling, we hypothesized that varying doses of PEF will modulate both Ca^2+^ levels and mechanical function of isolated cardiac myocytes. Gaining knowledge of EP effect on contractility and Ca^2+^ is becoming more relevant as PFA is now in clinical development and is speculated to become the preferred energy modality for cardiac ablation.^,20,21,40,41,42,43^

Thus, the goal of this study was to characterize reversible and irreversible effects of high voltage PEF on isolated left ventricular myocyte’s (LVM) contractile function, Ca^2+^ transient (CaTr) and recovery or cell death over a clinically relevant time frame (generally 20 minutes).^44^ Studied variables included pulse parameters (voltage, pulse width), pacing, as well as cell orientation in the applied electric fields.

## Methods

The data that support the findings of this study are available from the corresponding author upon reasonable request. All experiments were performed in accordance with the European Union council directive 2010/63/EU for the protection of animals used for scientific purposes following local ethical committee approval.

### Isolation of Rat Ventricular Myocytes

Male Wistar rat (250–350 g) were anesthetized (Isoflurane, 2.5%–3%) and euthanized. Hearts were quickly excised, mounted on a Langendorff system, and perfused at 8 mL/min (37 °C) through the aorta with an oxygenated isolation solution containing (in mM): 130 NaCl, 5.4 KCl, 1.4 MgCl_2_, 0.4 NaH_2_PO_4_, 5 HEPES, 10 Glucose, 10 Creatine, 20 Taurine (pH 7.4 with NaOH), and 0.75 mM CaCl_2_. The hearts were then perfused with the isolation solution supplemented with 0.1 mM Ethyleneglycol- bis(β-aminoethyl)-N,N,N′,N′-tetraacetic Acid (EGTA) and finally with the same solution with the addition of collagenases (type II, 1 mg/mL; Worthington) and proteases (type XIV, 0.1 mg/mL; Sigma Aldrich) fwor 8 to 10 minutes. The left ventricles were then dissected, cut into small pieces and gently shaken at 37 °C in the enzymatic solution. Myocytes were collected by filtration through a nylon gauze and were used within 8 hours.^45^

### Simultaneous Measurement of Sarcomere Length and Intracellular Ca^2+^

LVM were placed in a perfusion chamber mounted on the stage of an inverted microscope (Eclipse Ti-U, Nikon; France) and continuously perfused with a Tyrode solution (mM): 140 NaCl, 5 KCl, 1.0 CaCl_2_, 1 MgCl_2_, 10 glucose and 10 Hepes (pH 7.4 with NaOH) at 34 °C to 37 °C (TC2BIP; Cell MicroControls). Cardiomyocytes were paced via field-stimulation to contract at 1 Hz (ds2a field-stimulator, Digitimer Ltd, United Kingdom) and sarcomere length continuously monitored through a video-detection system (IonOptix Corporation). In some experiments, intracellular Ca^2+^ ([Ca^2+^]_i_) level was simultaneously monitored in myocytes loaded with 4 µM Fura-2 Fura-2 acetoxymethyl ester (Invitrogen) (Invitrogen). Briefly, following excitation at 340 and 380 nm, the emission light was collected at 510±40 nm through a photomultiplier tube connected to a spectrophotometer system (Cairn Research, United Kingdom). The ratio of the emitted light in response to 340 and 380 nm excitation was used as an index of [Ca^2+^]_i_. Sarcomere shortening expressed as a percentage of resting sarcomere length, diastolic intracellular Ca^2+^ level and CaTr amplitudes were analyzed using IonWizard (IonOptix Corporation).^46^ For pacing-induced activity, all measurements were performed at steady state and the analysis was done on averaged trace of 10 consecutive beats.

### PEF Treatment Protocols

Only rod-shaped myocytes showing clear striations were used. When the role of cell orientation with respect to the electric field was studied, only cells showing axis angle with respect to electric field of 0±10° (parallel) and 90±10° (perpendicular) were used for the experiments. Myocytes were exposed to EP at various voltage amplitudes using 2 platinum electrodes (4 mm spacing) connected to both a field-stimulator and a PEF generator (Electro Cell B10, Leroy Biotech, France) producing an electric field used for either pacing and electroporation, respectively.^47^ Electric field intensity (E) estimated as a voltage-to distance ratio and given in V/cm is provided for easier comparison. PEF were applied as a single monophasic EP of 100 µs duration at low (80 V [E=200 V/cm]), medium (140 V [E=350 V/cm]) voltage. At these field strengths, both contractility and [Ca^2+^]_i_ were monitored independently of cell orientation relative to the PEF. High-sublethal voltage EP (100 µs duration) were applied either between 300 and 440 V (E=750–1100 V/cm) to cells oriented parallel to the electric field or between 140 and 200 V (E=350–500 V/cm) to cells oriented perpendicular while monitoring contractility and [Ca^2+^]_i_. Pacing was stopped just before the EP application, and myocytes were left unpaced for 1 minutes after EP delivery. Sarcomere length and [Ca^2+^]_i_ were monitored during this unpaced period. After 1 minute, myocyte pacing was resumed to assess contractile and [Ca^2+^]_i_ recovery. Then, pacing was stopped and resumed every 5 minutes over a 20-minute period to monitor the presence of spontaneous contractions (Figure S1 for protocol design). Cells that recovered were then exposed to voltages of increasing intensity to identify the lethal voltage values. Another set of experiments was conducted in which EP was delivered under continuous pacing at 1 Hz. During this investigation, both sarcomere shortening and [Ca^2+^]_i_ parameters were evaluated every 5 minutes over a 20-minute period (Figure S2). We established the lethality profile by applying a single 10 ms EP at different voltage amplitudes (50–140 V). A complementary metal oxide semiconductor camera connected to the side port of the microscope (Imaging-S IMX265LQR, Imaging Source, Germany) was used to image isolated cardiac myocytes within the field of view prior and ≈30 s after EP application. The percentage of rod-shaped striated cells which died upon EP application (as determined by morphological assessment) was calculated separately for parallel- and perpendicular-oriented myocytes with respect to the electric field.

### Statistics

Experimental data are expressed as means±SEM; n represents the number of experiments; N represents the number of animals used for the experiments. Samples were tested for normal distribution with d’Agostino-Pearson. Statistical analysis was performed using GraphPad Prism version 6.0 (GraphPad Software, San Diego, California). Unpaired *t* test, paired *t* tests, or repeated-measure 1-way ANOVA or their respective nonparametric tests were used to compare our conditions. Differences were considered significant for *P*<0.05.

## Results

### Effect of Low- and Intermediate-Voltage PEFs on Left Ventricular Myocyte Contractility and [Ca^2+^]_i_

**Figure 1.**
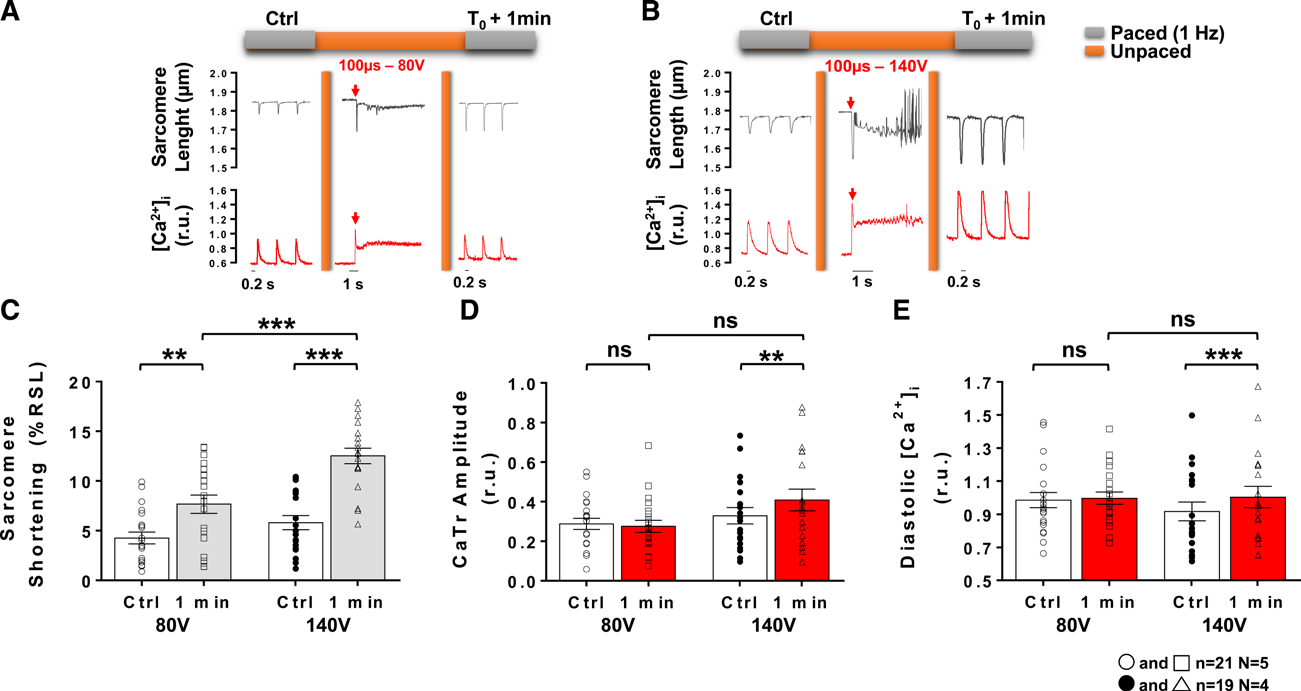
**Effects of low- and intermediate-voltage pulsed electric fields on left ventricular myocyte shortening and intracellular Ca2+ ([Ca2+]i) at 1 minute after application.** Representative sarcomere lengths and [Ca^2+^]_i_ traces obtained from an isolated left ventricular myocytes during baseline (control [Ctrl]) 1 Hz pacing and 1 minute after (T_0_+1 minute) a 100 µs electric pulse (EP) delivered at low- (80 V; **A**) and intermediate-voltage (140 V; **B**). Sarcomere shortening, expressed as a percentage of the resting sarcomere length (%RSL) was increased 1 minute after EP with a low- and an intermediate-voltage pulses (**C**). Ca^2+^ transient (CaTr) amplitudes (**D**) and diastolic Ca^2+^ levels (**E**) expressed in 340:380 ratio units (r.u.), were increased 1 minute after EP with an intermediate but not with a low-voltage pulse. Data are represented as mean±SE of the mean with individual values for each cell. N indicates number of animals; n, number of cells; and ns, not significant. Paired *t* test: ns, **P<0.01, ***P<0.001.

**Figure 2.**
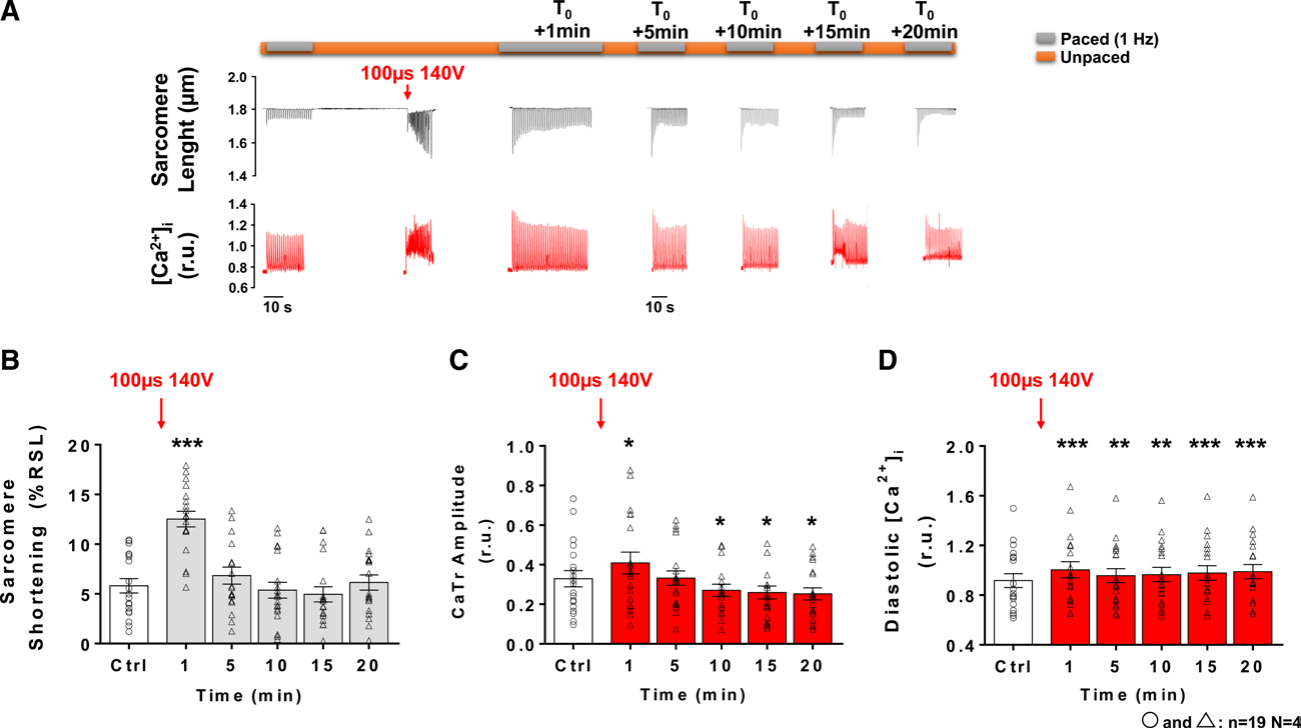
**Reversibility of sarcomere shortening and Ca^2+^ handling following exposure to intermediate voltage pulsed electric fields. A**, Representative sarcomere lengths and intracellular Ca^2+^ ([Ca^2+^]_i_) traces obtained from an isolated left ventricular myocytes (LVM) before (control [Ctrl]), during and 1 to 20 minutes after (T_0_+1 minute to T_0_+20 minutes) application of a 100 µs, 140 V pulsed electric field. **B**, Sarcomere shortening of LVM (expressed as a percentage of resting sarcomere length, %RSL) paced at 1 Hz increased 1 minute after electric pulse (EP) and returned to Ctrl level from 5 minutes to 20 minutes post-EP (**C**). Ca^2+^ transient (CaTr) amplitude was significantly increased at 1 minute and returned to Ctrl level at 5 minutes after EP. From 10 to 20 minutes after pulse application CaTr amplitude further decreased below Ctrl level. **D**, Diastolic Ca^2+^ level increased 1 minute after pulse delivery and remained higher than Ctrl level for the duration of the protocol. Data are represented as mean±SE of the mean with individual values for each cell. Repeated-measure 1-way ANOVA: *P<0.05, **P<0.01, ***P<0.001. N indicates number of animals; n, number of cells; and r.u., ratio units.

Typical sarcomere length and [Ca^2+^]_i_ traces are presented in Figures 1A and 1B prior to, during and 1 minute after the EP application of a 100 µs pulse with low- (80 V) and intermediate- (140 V) voltage amplitudes, respectively. In this context, the EP induced an immediate increase in [Ca^2+^]_i_ reaching higher level than systolic [Ca^2+^]_i_ observed when pacing at 1 Hz resulting in a large sarcomere shortening. At low-voltage amplitudes, the first peak CaTr recorded after EP was similar in amplitude to the first transient obtained upon resuming field-stimulation after an unpaced period and was reminiscent of postrest potentiation.^48^ At intermediate-voltage amplitudes, the first CaTr after EP was slightly greater than postrest first CaTr (Figure S2). This PEF-induced immediate increase in [Ca^2+^]_i_ was followed by a fast decrease in [Ca^2+^]_i_ accompanied by sarcomere relaxation similar to that observed upon pacing LVM. However, this relaxation was incomplete and [Ca^2+^]_i_ remained elevated for several seconds before returning closer to initial diastolic levels within 1 minute and beyond (Figures 1 and 2). At 1 minute post-EP, when 1 Hz pacing was resumed, a significant increase in sarcomere shortening was observed, which appeared to be dependent on the voltage amplitude of the pulse (Figure 1C). At low voltage, no significant change in CaTr amplitude (Figure 1D) or diastolic Ca^2+^ level (Figure 1E) could be observed. However, exposure to intermediate voltage amplitudes led to a significant increase in both these parameters (Figures 1D and 1E). The reversibility of the contractility and [Ca^2+^]_i_ changes induced by an intermediate voltage pulse was then assessed over a 20-minute time period by intermittently pacing myocytes at 1 Hz every 5 minutes (Figure 2). Potential time-dependent effects were first ruled out by sarcomere length and monitoring [Ca^2+^]_i_ over a 20-minute period in LVM not exposed to an EP (Figure S3). Representative traces showing sarcomere length and [Ca^2+^]_i_ traces at different times after exposure to an intermediate voltage amplitudes are shown on Figure 2A. The increase in sarcomere shortening and CaTr amplitude observed at 1 minute after EP returned to pre-EP levels at 5 minutes (Figures 2B and 2C). A significant decrease in CaTr amplitudes was observed at 10, 15 and 20 minutes after EP (Figure 2C). Diastolic Ca^2+^ levels increased at 1 minute following exposure to 140 V and did not return to baseline level within 20 minutes (Figure 2D). Similar results were obtained with continuous electrical pacing at 1 Hz throughout the protocol’s duration (Figure S4) with the exception of sarcomere shortening being significantly reduced 10, 15, and 20 minutes after EP.

### High-Voltage PEFs Differentially Affect LVMs Depending on Their Orientation With Respect to Electric Field

**Figure 3.**
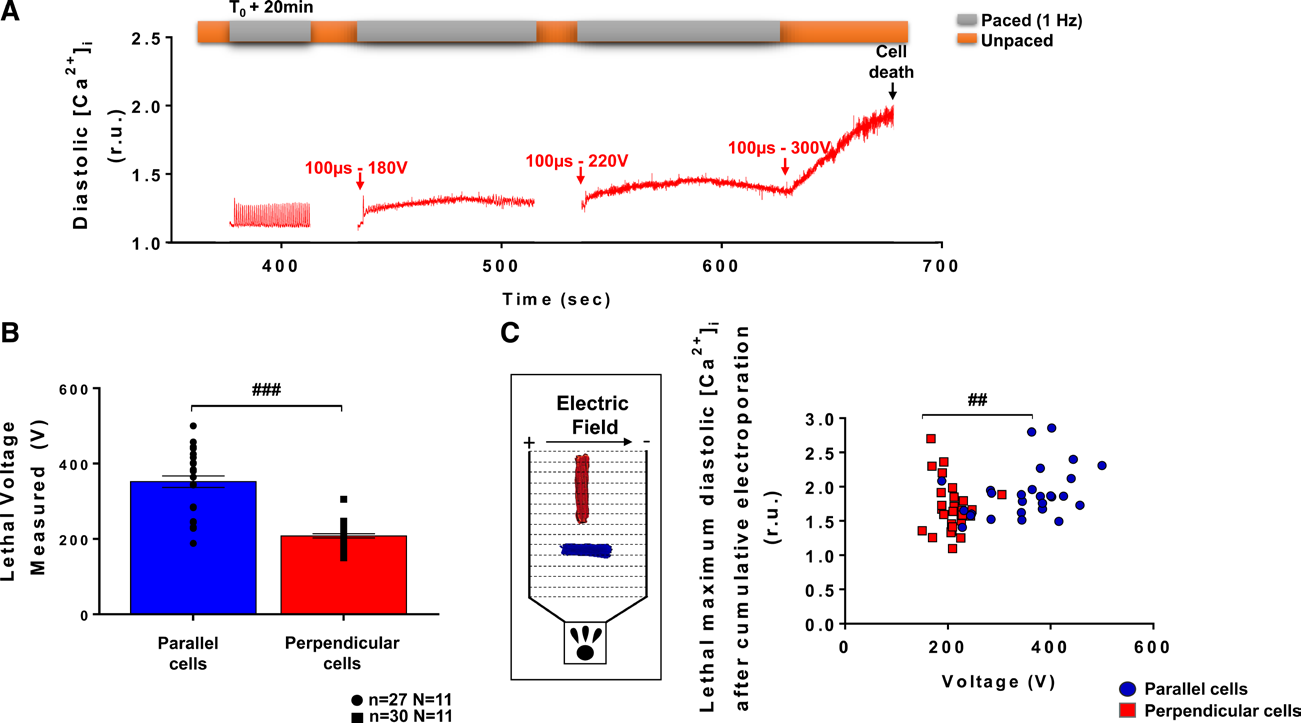
**Voltage threshold for irreversible electroporation depends on myocyte orientation with respect to the electric field.** Following an intermediate voltage pulse application (140 V, 100 µs), left ventricular myocytes (LVM) oriented parallel or perpendicular to the electric field were exposed to electric pulse (EP) of increasing voltage amplitudes until cell death was observed. **A**, Representative intracellular Ca^2+^ ([Ca^2+^]_i_) traces obtained in a LVM oriented parallel to the electric field show the rise in diastolic Ca^2+^ when incrementing voltage amplitudes. **B**, Lethal voltage thresholds differed according to cell orientation and with Fura-2. Data are represented as mean±SEr of the mean with individual values for each cell. **C**, Lethal diastolic Ca^2+^ levels in perpendicular cells were reached at lower voltages than in parallel-oriented cells. Inset: cell orientation and electric field direction. Unpaired *t* test: ##*P*<0.01; ###*P*<0.001. N indicates number of animals; n, number of cells; and r.u., ratio units.

**Figure 4.**
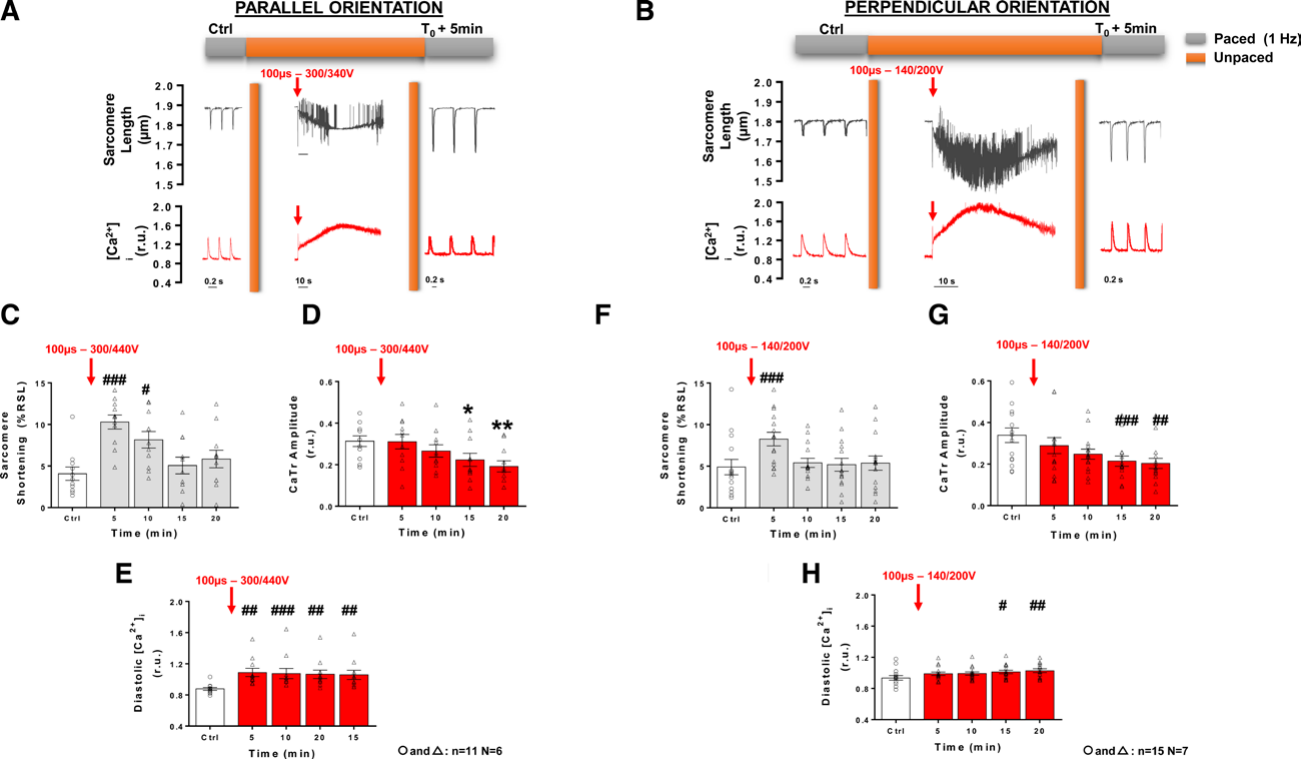
**Effect of high-voltage pulsed electric field on sarcomere shortening and intracellular Ca2+ ([Ca2+]i) in left ventricular myocytes according to cell orientation.** Representative sarcomere lengths and [Ca^2+^]_i_ traces obtained from an isolated left ventricular myocytes (LVM) during baseline (control [Ctrl]) 1 Hz pacing, during and after (T0+5min) a 100 µs high voltage electric pulse (EP) delivered to a parallel (**A**) and perpendicular-oriented cell (**B**) below their respective lethal threshold. Sarcomere shortening, expressed as a percentage of resting sarcomere length (%RSL) was increased 5 minutes after EP and returned to Ctrl level 15 minutes post-EP in parallel-oriented cells (**C**). Ca^2+^ transients (CaTr) amplitude progressively decreased after EP (**D**) while diastolic levels increased (**E**). Similar trends on sarcomere shortening (**F**), CaTr amplitudes (**G**) and diastolic Ca^2+^ levels (**H**) were observed in perpendicular-oriented cells. Data are represented as mean±SE of the mean with individual values for each cell. Repeated-measure 1-way ANOVA: *P<0.05, **P<0.01, #*P*<0.05, ##*P*<0.01, ###*P*<0.001. N indicates number of animals; n, number of cells; and r.u., ratio units.

After exposing LVM to low- and intermediate-voltage amplitudes, we then applied EPs of increasing voltage amplitude to determine the voltage threshold at which myocytes were irreversibly electroporated. This experiment is illustrated in Figure 3A. Twenty minutes after a first pulse delivery (100 µs, 140 V), myocytes partially recovered (as seen in Figure 2) and were then subjected to several pulses of increasing amplitude. Lethal effect, as determined by a loss of LVM morphological integrity (ie, shrinking from rod-shaped to round-shaped myocytes with loss of clear striations), was achieved at lower voltages in perpendicular cells than in parallel cells (Figures 3B and 3C). The diastolic Ca^2+^ levels found in cells for both orientations at lethal voltages were significantly different (Figure 3B). Similar, albeit somewhat higher) lethal thresholds were also obtained with a different protocol consisting of applying a single monophasic EP to the cell (Figure S4), that is, avoiding potential cumulative effect of multiple pulses being applied to the same cells. We also assessed the effect of longer-lasting electroporating pulses on lethality in both perpendicular and parallel-oriented myocytes with respect to electric field. Quiescent myocytes were exposed to single 10 ms electroporating pulses of different voltage amplitudes and lethality was assessed by image analysis (Figure S6A). Interestingly, for this pulse duration, parallel myocytes were more sensitive to PEF-induced cell death than perpendicular cells (Figure S6B and Table S1). This led us to assess the effect of high voltage, sublethal PEF on contractility and [Ca^2+^]_i_ at distinct voltage amplitudes for these 2 cell subpopulations, that is, 300 to 340 V for cells oriented parallel and 140 to 200 V for cells oriented perpendicular to the electric field. At 1 minute post-EP with high voltage, sublethal pulses, 60% of perpendicular, and 55% of parallel cells presented elevated diastolic [Ca^2+^]_i_ level and remained unresponsive to pacing. In cells that responded to pacing at 1 minute post-EP, a significant increase in sarcomere shortening was observed in perpendicular but not in parallel myocytes following 1 minute after EP (Figure S7A and S7B). At this time, high voltage pulses did not significantly alter CaTr amplitudes in either cell groups but there was a trend for an increase in diastolic Ca^2+^ level in both parallel and perpendicular myocytes (Figure S7). At 5 minutes after applying a high voltage amplitude pulse, a significant increase in sarcomere shortening was observed in LVM for both orientations (Figures 4A, 4B, 4C and 4F), despite no change in CaTr (Figures 4D and 4G). This increase in sarcomere shortening returned to baseline after 10 and 15 minutes in perpendicular and parallel cells, respectively, and remained stable until 20 minutes after EP. As seen with intermediate voltage pulses, a progressive decrease in CaTr amplitude was found in both parallel and perpendicular cells between 5 and 20 minutes after pulse application. High voltage pulses induced a significant increase in diastolic Ca^2+^ levels at 5 minutes in parallel-oriented myocytes which remained constant within 20 minutes post-EP (Figure 4E). In myocytes oriented perpendicular to the electric field, the increase in diastolic Ca^2+^ levels was progressive and only reached statistical significance at 15 and 20 minutes after EP (Figure 4H).

### PEF-Induced Alteration in Ca^2+^ Level and Spontaneous Contractile Activity

**Figure 5.**
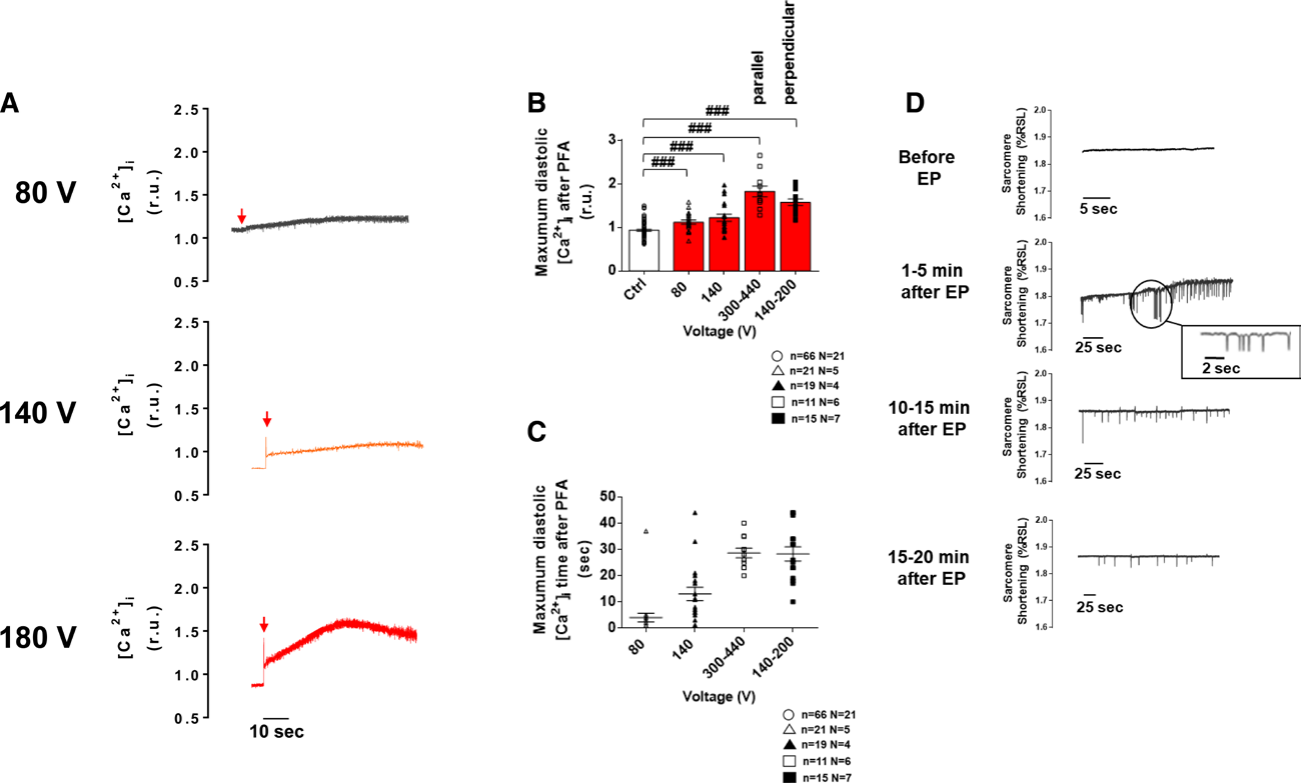
**Immediate effect of a monophasic single electric pulse (EP) on left ventricular myocyte diastolic Ca^2+^ levels and spontaneous contractile activity. A**, Representative intracellular Ca^2+^ ([Ca^2+^]_i_) traces from left ventricular myocytes (LVMs) subjected to a low- (80 V), medium- (140 V), and high-voltage (here, 180 V in a perpendicular cell) 100 µs EP. **B**, The rise in diastolic Ca^2+^ level induced by the EP increased when increasing pulse’s amplitude. **C**, The time to reach maximum diastolic Ca^2+^ level also tended to increase with the voltage of the electroporating pulse. **D**, Example of spontaneous contractions monitored before and after EP (intermediate voltage, 100 µs) in unpaced LVM. Prior to EP, when pacing was stopped, LV myocytes remained quiescent. Spontaneous contractions were observed in these cells 1–5 minutes after EP and their frequency decreased with time over 20 minutes. Data are represented as mean±SE of the mean with individual values for each cell. Unpaired *t* test: ###*P*<0.001. N indicates number of animals; n, number of cells; PFA, pulsed field ablation; and r.u., ratio units.

Independently of the voltage applied and of cell orientation relative to the applied field, a monophasic single EP application induced an instantaneous increase in [Ca^2+^]_i_ followed by a rapid decrease and either a plateau phase, for low- and intermediate-voltage pulses, or a slow increase in diastolic Ca^2+^ for high voltage pulses (Figure 5A). Interestingly this is consistent with transiently increased membrane permeability described in electroporation for Ca^2+^ and/or other ions and molecules. The maximum increase in diastolic Ca^2+^ levels showed dependency on EP voltage amplitude with higher voltages leading to greater diastolic Ca^2+^ accumulation (Figure 5B and 5C). It must be noted that during this time of increased Ca^2+^, myocytes were not responsive to electrical pacing pulses. This increase in diastolic Ca^2+^ levels was followed by spontaneous Ca^2+^ events (not shown) and contractions which progressively disappeared with time whatever the voltage (Figure 5D).These spontaneous events were only observed in reversibly electroporated myocytes. In myocytes exposed to IRE, intracellular Ca^2+^ continued to rise after PEF application until cell death.

## Discussion

This study provides new insights into the effects of PEF on LVM function. We determined for the first time the influence of PEF on myocyte contractile function and [Ca^2+^]_i_ over a clinically relevant time scale (20 minutes). This constitutes an important step to better understand mechanisms involved in cardiac PFA. As clinical ablation therapies based on the principles of IRE are being developed, it is important to understand the underlying cellular effects of irreversible but also of reversible electroporation in cardiac myocytes.

### Effects of Sublethal EPs on Ventricular Myocyte Excitation-Contraction Coupling

EP is being exploited in a large spectrum of biological discoveries and clinical treatments. Reversible electroporation is used for gene delivery^49^ or enhancing chemotherapy effectiveness,^6^ whereas IRE is used in treatments involving oncology^6^ and cardiac ablation domains.^,14,21,43,50^ However, it must be noted that even when IRE is used, electric field attenuation within the tissue as a function of distance to the electrodes may lead to reversible effects in the adjacent myocardium.

It is well established that the cardiac contractility is modulated by a specific equilibrium of intracellular Ca^2+^.^51^ Thus, the process of excitation-contraction coupling is important because it is at the interface of the electrical membrane activity (action potential) and cell contraction.^52^ Normal heart function requires an accurate regulation of [Ca^2+^]_i_ to ensure fine-tuned diastolic (low [Ca^2+^]_i_) and systolic (high [Ca^2+^]_i_) cycles in normal heart function. In our study, we found that EPs perturbed intracellular Ca^2+^ homeostasis. First, we found an elevated [Ca^2+^]_i_ plateau which followed EP application before returning to lower diastolic levels in LVM exposed to low-, intermediate-, and high-sublethal voltage EPs. This intracellular accumulation of Ca^2+^ depended directly upon EP voltage amplitudes and was associated with cell death when subsequent PEF of increasing amplitude were applied to myocytes. Such a PEF voltage-dependent effect was likely related to an increase in sarcolemma pore density which is known to increase with increasing electric field strengths.^38^ The degree of membrane (electro)permeabilization is a key factor determining the fate of the cell after exposure to high voltage amplitudes; either cell death (IRE) or recovery (reversible electroporation). Indeed, this physiological process (cell recovery) is crucial to maintain cellular function and to preserve the integrity of the plasma membrane as well as the Ca^2+^ handling machinery^53–55^ to limit undesirable exchange of ions and other components between intracellular and extracellular media. Here, we could not determine the Sarco/endoplasmic calcium ATPase (SERCA) or sodium-calcium exchanger (NCX) function in myocytes immediately after EP application as these would not respond to pacing at this point. However, at 1 minute after EP—or 5 minutes for high voltage EPs—the CaTr decay time constant, an index of function of Ca^2+^ removal pathways, was similar to that measured prior to EP for all sublethal voltages. It has been previously shown that the recovery times of cells/tissues exposed to electroporating pulses depend mainly on the pulse settings (e.g. waveform, voltage), the cell orientation and the experimental buffer.^,34,39,56^ Note that myocytes partially recovered their capacity to normally respond to the pacing as early as 1 minute after EP for low- and intermediate-voltage amplitudes. However, after this time, we also identified spontaneous contractions which were associated with spontaneous intracellular Ca^2+^ releases and elevated diastolic Ca^2+^ levels. Those spontaneous contractions were more frequent when myocytes were exposed to high voltage EP, indicative of a possible membrane defect (cell membrane and/or sarcoplasmic reticulum). Abnormal intracellular Ca^2+^ responses have been described upon exposing mouse ventricular myocytes to repetitive nanoseconds pulse-width PEFs.^37^ In our study, we observed these events already after the application of a single monophasic EP, but these may be expected also after a train of pulses being delivered. Further work will be required to confirm the effect of PEFs on Ca^2+^ homeostasis at the tissue level in coupled myocytes and evaluate their arrhythmogenic potential. To our knowledge, no arrhythmic event has been reported from clinical and preclinical studies using PFA. We can speculate that the large increase in diastolic Ca^2+^ level induced by PEFs may induce a closure of the connexins and reduce cell-to-cell coupling until intracellular Ca^2+^ levels are regulated. This would prevent the propagation of the spontaneous arrhythmogenic Ca^2+^ events to the surrounding myocardium. It must be noted that EP appeared to be reversible (20 minutes after EP) and may thus indicate the cells’ ability to regulate their Ca^2+^ homeostasis to maintain their function. Interestingly 5 to 20 minutes is also time reported for cell membrane resealing in electroporation in vitro.^,35,58,39,57^ Previous studies demonstrated similar time courses of recovery after the exposure to high-voltage amplitudes.^25,35,39,57,59–61^ Here, despite an initial increase in sarcomere shortening and CaTr upon resuming pacing, there was a trend for a decrease in these parameters below their control level and a rise in diastolic [Ca^2+^]i within a 20-minute time frame after EP. These results suggest myocytes may not completely recover from PEF application within the observed 20 minutes although further experimental investigations are required to evaluate myocyte recovery in the longer term.

### Influence of Cell Orientation With Respect to the Electric Field on Myocyte IRE

Irreversible electroporation, resulting in cell death, caused by electroporating pulses is mainly related to increased permeabilization of the plasma membrane which occurs when the membrane potential reaches supraphysiologic values. Indeed, when the membrane potential exceeds a critical value, a disruption of the plasma membrane structure can trigger a detrimental biological process which is manifested by the formation and expansion of large hydrophilic pores, massive Ca^2+^ influx, mitochondrial damage leading to ATP depletion, reactive oxygen species production, and an increase of the cell content leakage inexorably leading to cell death.^35,62–66^

Interestingly, when applying 100 µs pulses, we observed that cells oriented perpendicular were more sensitive to the PEF-induced cell death than cells oriented parallel to the electric field. The elongated geometry of LVM may contribute to this effect as suggested in silico by others.^67^ The specific membrane structure of LVM with transverse tubules may also contribute although this will require further investigations. However, when applying 10 ms pulses the opposite effect was observed, that is, cells oriented parallel were more sensitive to PEF-induced cell death then cells oriented perpendicular to the electric field. Parallel cells being more sensitive than perpendicular is in line with previous study in which authors also identified a lower lethal threshold in ventricular myocytes oriented parallel to the electric field using 10 ms pulses.^,35,39^ For millisecond pulses, it has been suggested this could be related to the fact that the maximum change in membrane potential occurs at the cell ends which are close to the electrodes and to the threshold for electroporation which is proportional to the cell length in the direction of the field.^38^

A similar change in sensitivity of cells to PEF according to their orientation has recently been unveiled in cardiac-like cell lines, where short and long pulses had opposite effects, a similar observation as in our study—although the crossover pulse duration appeared to happen at shorter pulse durations.^38^ This discrepancy may be related to the different type of cells and their respective geometries and membrane properties. The mechanisms underlying this different sensitivity to EP in parallel and perpendicular cells according to pulse duration are not well explained but may involve differences in the way pores are formed^37^ or membrane curvature.^67^ Identifying the pulse parameters for which parallel and perpendicular cells share a similar sensitivity to IRE, may facilitate the creation of more homogeneous lesions within the thickness of tissues with heterogeneously-oriented cells such as in the atrial wall.

### Limitations

The study was conducted on isolated rat LVM. Different cardiac cell types (e.g. atrial, conduction system) or species (large animal, human) could be associated with different effects. For example, atrial myocytes and Purkinje cells are known to have different structures with less transverse tubules compared with ventricular myocytes.^,68,69^ In order to carefully identify the effects of PEFs on cardiac myocytes we used monophasic single pulses of varying duration and amplitude. However, clinical IRE protocols typically involve biphasic train of pulses. These protocols may require higher field strength as suggested by former studies in noncardiac cell lines.^,70,71^ The mechanisms by which PEFs affect Ca^2+^ homeostasis and contractility described in the present study are likely to be similar. Furthermore, it is also well established that there are physiological or pathological ionic current remodeling and Ca^2+^ handling changes according to the species and age that may influence PEF-induced Ca^2+^ changes and interpretations.^72,73^

### Conclusions

We identified reversible effects of PEF on isolated LVM excitation-contraction coupling during a clinically relevant time scale and defined the impact of Ca^2+^ changes in IRE myocyte. Myocyte capacity to recover from a monophasic PEF was time-dependent and was related to pulse voltage and duration. Irreversible effects, that is, cell death, involved a large increase in [Ca^2+^]_i_ and strongly depended on myocyte orientation within the electric field. Further work is however necessary to expand the knowledge from monophasic single pulses of 100 µs and 10 ms pulses used in our study to multiple pulse frequencies and biphasic waveforms on cardiac myocytes.

## Article Information

### Acknowledgments

We thank Celine Ayez for taking care of the animals.

### Sources of Funding

This study was funded by Medtronic.

### Disclosures

Drs Sigg and Stewart are employees of Medtronic. Dr Miklavčič is a Medtronic consultant. Drs Batista Napotnik and Miklavčič received a Medtronic grant. The other authors report no conflicts.

### Supplemental Material

Table S1

Figures S1–S4

## Supplementary Material


