# Supplementary material for: Reversible and Irreversible Effects of Electroporation on Contractility and Calcium Homeostasis in Isolated Cardiac Ventricular Myocytes
Source: Circ Arrhythm Electrophysiol. 2022 Oct 28;15(11):e011131. doi: 10.1161/CIRCEP.122.011131 (PMC9665944; doi:10.1161/CIRCEP.122.011131)
Supplement: Supplementary file 1 [file hae-15-e011131-s001.pdf]

SUPPLEMENTAL MATERIAL

	Parallel to the electrical field			Perpendicular to the electrical field		
Voltage (V)	0 ± 10°	n	N	90 ± 10°	n	N
140	100 ± 0.00	43	2	87.5 ± 5.94	32	2
120	92.31 ± 4.00	39	4	65.93 ± 5.00	91	4
100	82.86 ± 4.54	70	4	38.16 ± 5.61	76	4
90	75.00 ± 5.64	60	4	32.10 ± 5.22	81	4
80	46.15 ± 8.09	39	4	3.85 ± 2.69	52	4
70	27.27 ± 6.06	55	4	6.58 ± 2.86	76	4
60	10.77 ± 3.87	65	4	0.00 ± 0.00	84	4
50	3.08 ± 2.16	65	4	1.35 ± 1	74	4

Table I: Myocyte lethality following a single 10 ms PEF application according to the cell orientation. Only cells showing axis angle with respect to electric field of 0 ± 10° (parallel) and 90 ± 10° (perpendicular) were used for this experiment. Lethality was established by the loss of morphological integrity and expressed as a percentage of the total number of cells for a given orientation. n: number of cells, N: number of animals.

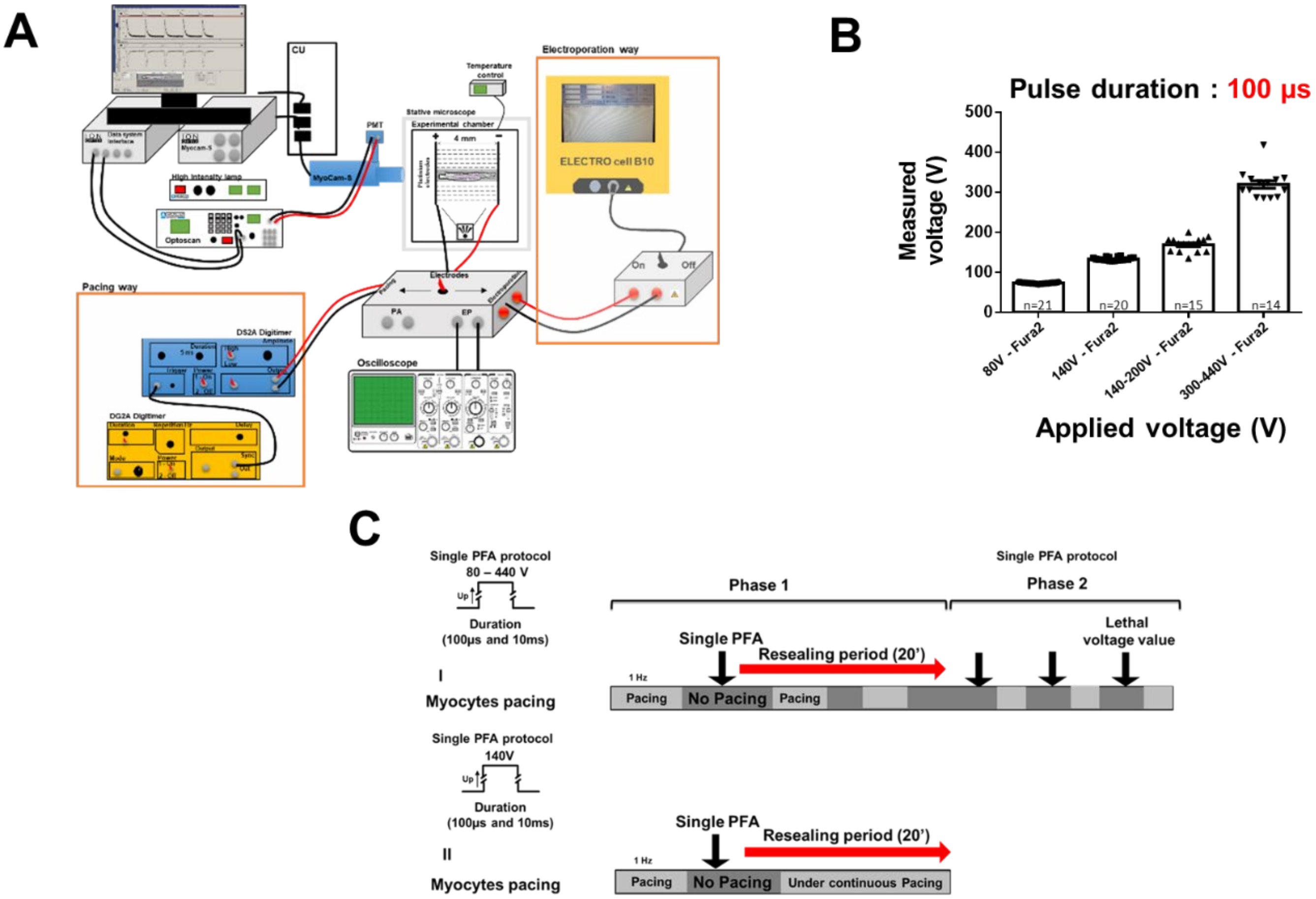

Figure I: Schematic representation of the experimental set up and quality control results. (**A) Experimental set up.** Cardiomyocytes were field-stimulated to contract at 1 Hz (DS2A stimulator, Digitimer Ltd, UK) and electroporated (Electro Cell B10, Leroy Biotech, France) in an experimental chamber using 2 platinum electrodes (4 mm spacing). All experiments were performed at 34 - 37°C. Sarcomere shortening and Ca^{2+} transients were recorded by video contrast analysis (IonOptix Corporation, USA) and spectrofluorimetry (Cairn Research, UK) in Fura-2 AM loaded (4 µM) myocytes. (**B) Quality control of the voltage delivered by the EP generator**. The voltage delivered during electrical pulses was monitored using an oscilloscope to confirm the generator operated at the requested voltage (V). n: number of cells - data are mean ± standard error of the mean. Dots in bar graphs represent individual values. **(C) Electroporation protocols.** Different protocols were used to determine the effect of single monophasic electroporating pulse of varying duration/amplitude on myocyte contractility, $[Ca^{2+}]_i$ and evaluate cell recovery and lethality.

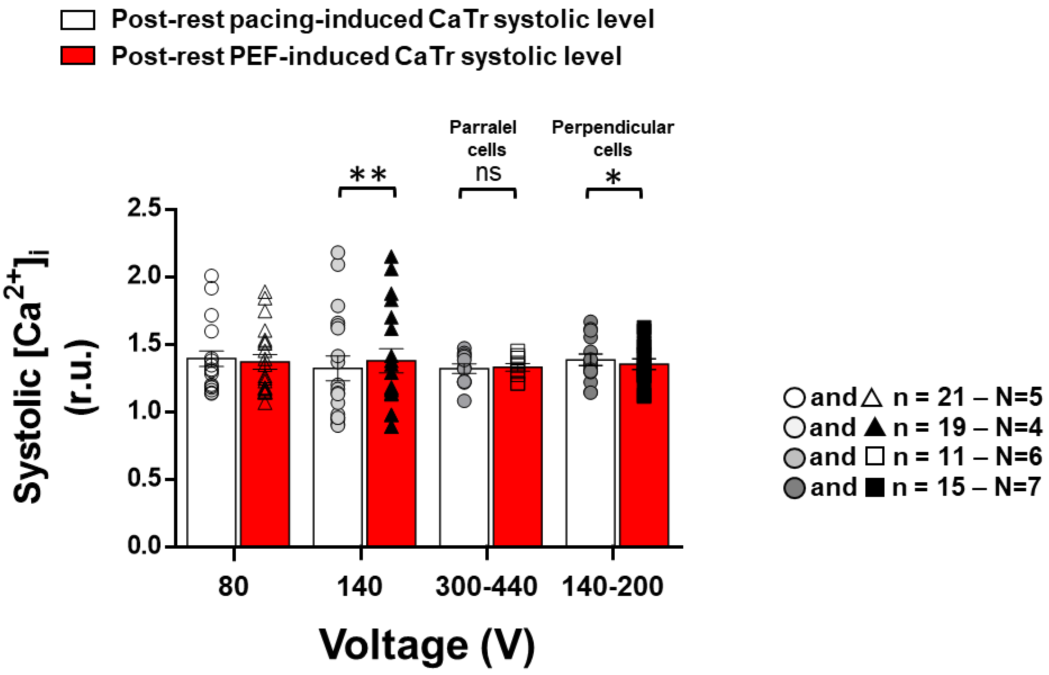

Figure II: Comparison of pacing-induced versus PEF-induced post-rest systolic $[Ca^{2+}]_i$. Prior to EP, quiescent left ventricular myocytes were electrically-stimulated to contract at 1 Hz. The amplitude of the first Ca^{2+} transient upon initiating 1 Hz pacing (open bars) was compared to that of the Ca^{2+} transient induced by an electroporating pulse in the same cell (red bars). Data are represented as mean ± standard error of the mean with individual values for each cell. Paired t-test: *: ns: not significant, $p<0.05$, **: $p<0.01$. n: Number of cells; N: number of animals.

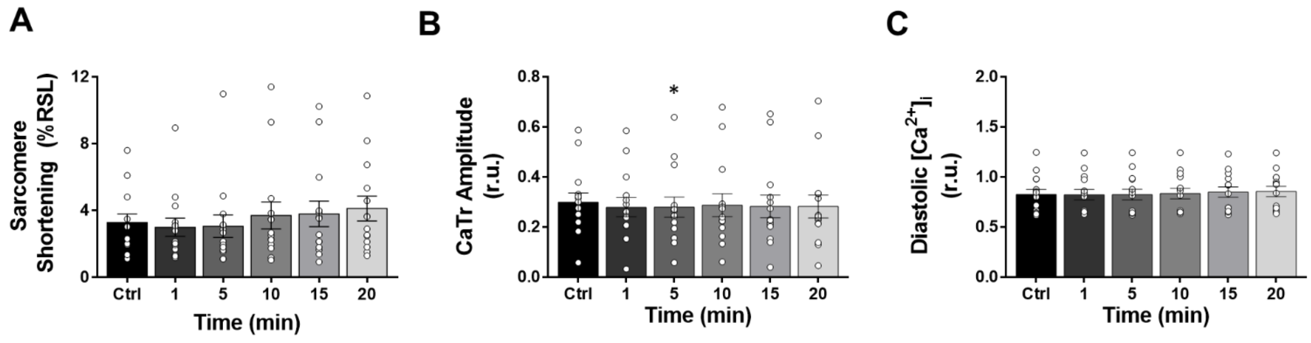

n=14 N=5 with fura2

Figure III: Time-related effects on rat left ventricular myocyte contractility and $[Ca^{2+}]_i$. Myocytes were subjected to the same pacing protocol (1 Hz) at 34-37°C but without EPs. **A:** Sarcomere shortening expressed as percentage of the resting sarcomere length (%RSL). **B:** Ca^{2+} transient (CaTr) amplitude expressed as a 340:380 fluorescence ratio (r.u., ratio units). C: Diastolic Ca^{2+} level remained stable across time. Data are mean ± standard error of the mean. Dots in bar graphs represent individual values RM on-way ANOVA *: $p<0.05$. n: number of cells, N: number of animals.

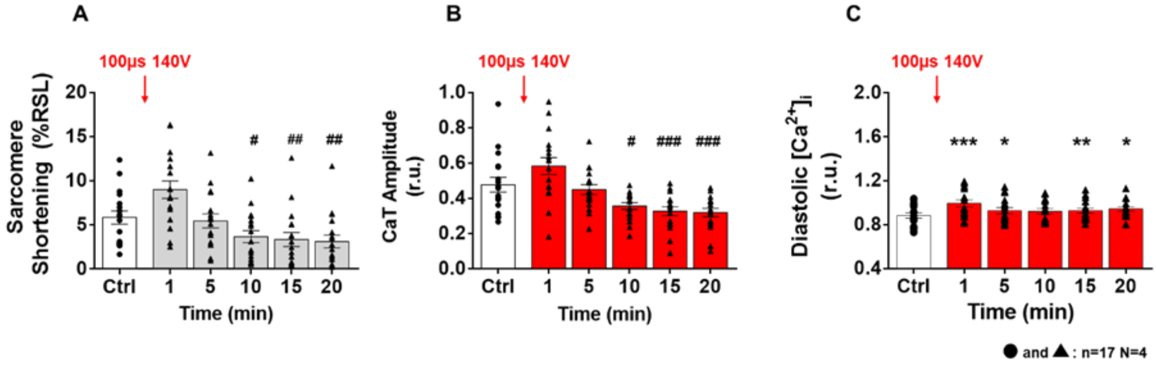

Figure IV: Reversibility of sarcomere shortening and Ca^{2+} handling following intermediate voltage electroporation under continuous electrical pacing. Sarcomere lengths and $[Ca^{2+}]_i$ were monitored in isolated LVM before (Ctrl), during and 1 to 20 min after (T0+1min to T0+20min) a 100 µs EP delivered at 140V, in myocytes continuously paced at 1 Hz to contract. Sarcomere shortening (A), expressed as a percentage of resting sarcomere length (%RSL) and Ca^{2+} transient (CaTr) amplitude (r.u., 340:380 ratio units) (B) were significantly reduced at 10, 15, 20 min post- EP. Diastolic Ca^{2+} level was significantly increased after EP. Data are represented as mean ± standard error of the mean with individual values for each cell. RM one-way ANOVA: *: p<0.05, **: p<0.01, ***: p<0.001, #: p<0.05, ##: p<0.01, ###: p<0.001. n: number of cells, N: number of animals.

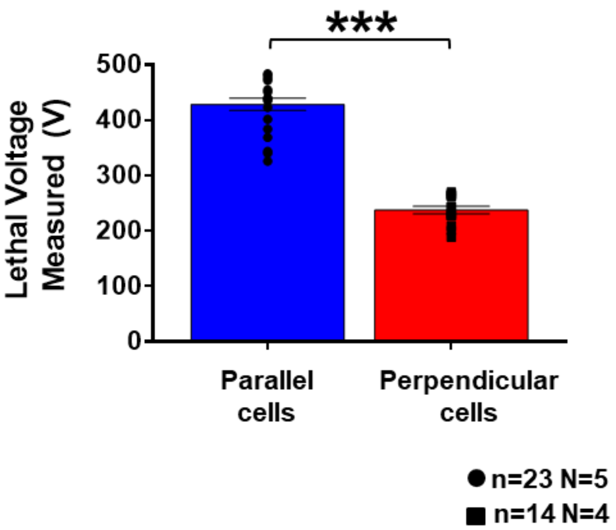

Figure V: Lethal voltage threshold following a single electroporating pulse application in parallel and perpendicular-oriented myocytes without Fura 2. Myocytes were subjected to a single 100 µs EP of varying voltages. Data are mean ± standard error of the mean. Dots in bar graphs represent individual values. Unpaired t-test: *** p<0.001. n: number of cells, N: number of animals.

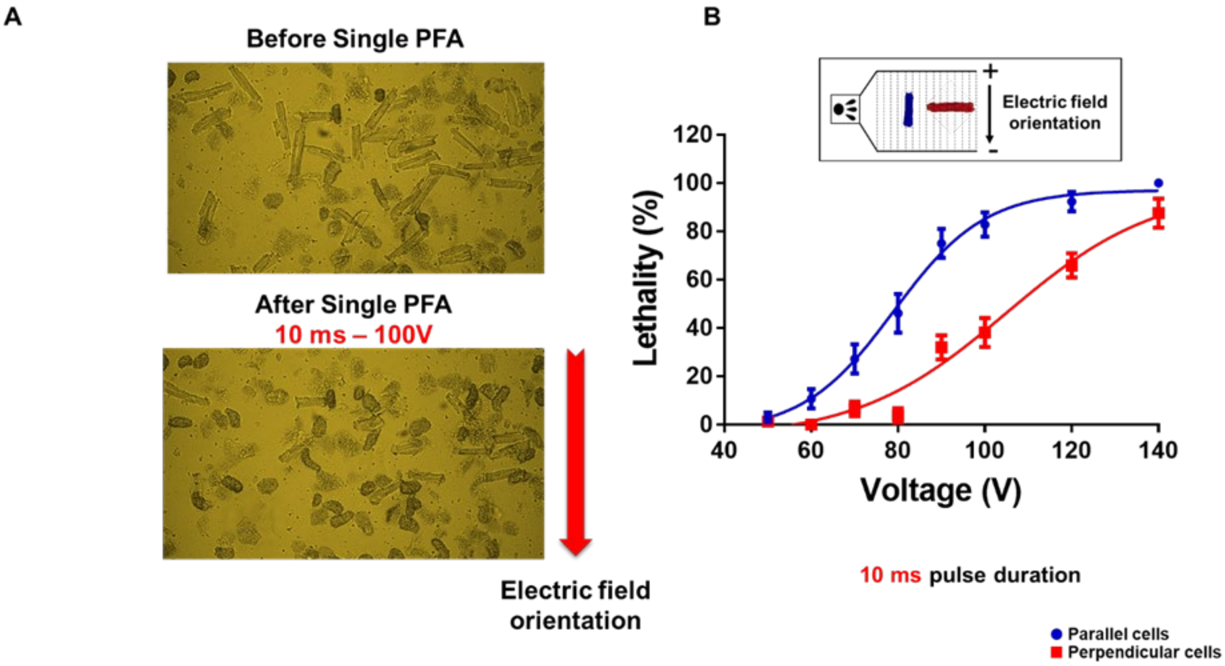

Figure VI: Lethal effect of a single 10ms electroporating pulse on left ventricular myocytes according to the cell orientation. (A) Images taken before and after a 10 ms, 100 V EP application were used to quantify lethality in LVM oriented parallel and perpendicular to the electric field. **(B)** Lethality curves representing the percentage of dead cells as a function of voltage and according to cell orientation. Cell death increased as the EP voltage was increased. Parallel cells (red) were more sensitive to PEF-induced cell death than perpendicular cells (blue) (voltage at 80% of lethality: 96 Vs 151 V for parallel and perpendicular cells respectively). Inset: cell orientation and electric field direction. Data are represented as mean ± standard error of the mean.

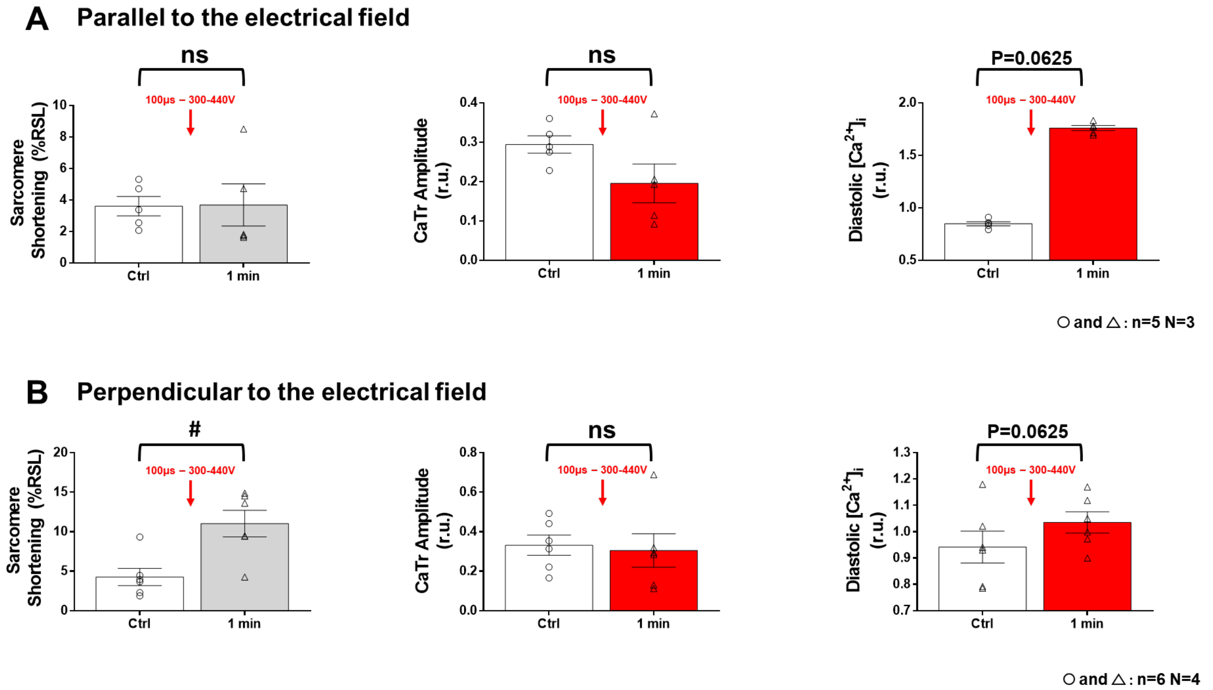

Figure VII: Effect of high voltage pulses on sarcomere shortening and $[Ca^{2+}]_i$ in left ventricular myocytes oriented parallel and perpendicular to the electric field at 1 min post-electroporation. Left ventricular (LV) myocyte sarcomere lengths and $[Ca^{2+}]_i$ were monitored before and 1 min after the application of a high voltage electroporating pulse (100 µs, 300-340 V for parallel, 140-200 V for perpendicular cells). Note that only 45% of parallel and 40% of perpendicular-oriented myocytes responded to 1 Hz pacing at 1 min post- EP. **A:** In parallel cells, sarcomere shortening (expressed as a percentage of resting sarcomere length (%RSL), Ca^{2+} ransient (CaTr) amplitude and diastolic Ca^{2+} level did not change significantly after EP despite a trend for an increase in diastolic Ca^{2+} level. **B:** In perpendicular cells, sarcomere shortening was significantly increased 1 min after EP while CaTr amplitude and diastolic Ca^{2+} level remained unchanged. Data are represented as mean ± standard error of the mean with individual values for each cell. Paired t-test: #: ns: not significant, p<0.05. Number of cells, N: number of animals.
